# Communication patterns in a psychotherapy following traumatic brain injury: A quantitative case study based on symbolic dynamics

**DOI:** 10.1186/1471-244X-11-119

**Published:** 2011-07-27

**Authors:** Paul E Rapp, Christopher J Cellucci, Adele MK Gilpin, Miguel A Jiménez-Montaño, Kathryn E Korslund

**Affiliations:** 1 Department of Military and Emergency Medicine, Uniformed Services University, 4301 Jones Bridge Road, Bethesda, MD 20814, USA; 2 Aquinas, LLC, 2014 St. Andrews Drive, Berwyn, PA 19312, USA; 3 Hunton and Williams LLP, 2200 Pennsylvania Ave. NW, Washington, DC 20037, USA; 4 Department of Epidemiology and Public Health, University of Maryland School of Medicine, Howard Hall, Suite 200, 660 W. Redwood Street, Baltimore, MD 20201 USA; 5 Facultad de Física e Inteligencia Artificial, Universidad Veracruzana, Sebastián Camacho #5, Col Centro, Xalapa, Ver. 91000, Mexico; 6 Department of Psychology, University of Washington, Box 355915, Seattle, WA, 98195, USA

**Keywords:** **traumatic **brain injury, psychotherapy, psychoanalysis, complexity, mutual information, entropy, information theory, symbolic dynamics

## Abstract

**Background:**

The role of psychotherapy in the treatment of traumatic brain injury is receiving increased attention. The evaluation of psychotherapy with these patients has been conducted largely in the absence of quantitative data concerning the therapy itself. Quantitative methods for characterizing the sequence-sensitive structure of patient-therapist communication are now being developed with the objective of improving the effectiveness of psychotherapy following traumatic brain injury.

**Methods:**

The content of three therapy session transcripts (sessions were separated by four months) obtained from a patient with a history of several motor vehicle accidents who was receiving dialectical behavior therapy was scored and analyzed using methods derived from the mathematical theory of symbolic dynamics.

**Results:**

The analysis of symbol frequencies was largely uninformative. When repeated triples were examined a marked pattern of change in content was observed over the three sessions. The context free grammar complexity and the Lempel-Ziv complexity were calculated for each therapy session. For both measures, the rate of complexity generation, expressed as bits per minute, increased longitudinally during the course of therapy. The between-session increases in complexity generation rates are consistent with calculations of mutual information. Taken together these results indicate that there was a quantifiable increase in the variability of patient-therapist verbal behavior during the course of therapy. Comparison of complexity values against values obtained from equiprobable random surrogates established the presence of a nonrandom structure in patient-therapist dialog (P = .002).

**Conclusions:**

While recognizing that only limited conclusions can be based on a case history, it can be noted that these quantitative observations are consistent with qualitative clinical observations of increases in the flexibility of discourse during therapy. These procedures can be of particular value in the examination of therapies following traumatic brain injury because, in some presentations, these therapies are complicated by deficits that result in subtle distortions of language that produce significant post-injury social impairment. Independently of the mathematical analysis applied to the investigation of therapy-generated symbol sequences, our experience suggests that the procedures presented here are of value in training therapists.

## Background

Traumatic brain injury is a significant cause of acute and long-term disability. Neurobehavioral sequelae encompass cognitive, social and psychiatric domains. Major depressive disorder is the most prevalent psychiatric disorder following traumatic brain injury regardless of the severity of the injury [1-9]. Estimates of prevalence are highly varied. Iverson, et al. [10] reviewed six studies of depression following traumatic brain injury and found reports of prevalence ranging from 12% to 44%. While prevalence rates are uncertain, a critical conclusion can be made. The treatment of neuropsychiatric disorders following traumatic brain injury is a significant clinical need that presents unique clinical challenges.

As commonly conceptualized, the clinical response to traumatic brain injury has four components: neuroprotection (preserve injured neurons), plastic modification (reconstruct neural networks with surviving neurons by promoting dendritic arborization and synaptogenesis), neurogenesis (stimulate the maturation of new neurons from stem cell populations), and neurointegration (facilitate the integration of newly formed neurons into the central nervous system). It is increasingly recognized, however, that psychotherapy is an important complement to this neurological response. Cope [11] has argued that "the majority of recovering survivors of TBI are now seen as potentially benefiting from some form of psychotherapeutic/rehabilitation treatment." Nonetheless, most individuals experiencing a head injury do not receive psychotherapy. In a review of the early history of psychotherapy following TBI, Prigatano [12] addressed the question, "Why has the role of psychotherapeutic interventions in the rehabilitation care of TBI patients gone unrecognized?" He suggests that "the answer seems to lie in the assumption that TBI patients could not benefit from psychotherapy because of their permanent cognitive, linguistic and affective disturbances." While this argument might be advanced when considering severe TBI, it does not seem plausible in cases of mild TBI. But is it even applicable in the case of severe TBI? Results reported by Ben-Yishay et al. [13] and by Ezrachi, et al. [14] indicate that psychotherapy following moderate or severe TBI has a positive effect on post-injury employment.

While psychotherapy is the preferred approach to the treatment of mood disorders following traumatic brain injury [1,2,15-17] there is limited research to help guide the selection of the specific therapeutic method [18,19]. The heterogeneity of this population demands a varied response. In part, the appropriate therapy will be determined by the physical injury, particularly the residual neurological and cognitive deficits. Individuals with TBI may benefit from treatments that take post-injury cognitive distortions into account [20-22]. The choice of therapy should also be responsive to pre-injury psychopathology [23,24]. There is an emerging literature detailing the benefits of cognitive behavior therapy across a variety of medical patients with acquired brain injuries of various severities comorbid with mood disorders [15-18,25,26]. Psychodynamic psychotherapy has also been considered. While cognitive deficits following head injury can limit the individual's ability to profit from psychodynamic psychotherapy, this is not invariably the case. As Lewis and Rosenberg [27] observed in a paper describing psychoanalytic psychotherapy following brain injury, "the overriding principle that guides such psychotherapeutic work is that acquired brain lesions do not ablate the patient's psyche or unconscious." These authors have identified five criteria that can help identify candidates for psychoanalytic psychotherapy following brain injury. (1.) The patient must be motivated to enter and remain in therapy. (2.) Patients who have had at least one positive significant relationship in the past are better able to form a therapeutic alliance. (3.) Patients who have had previous successes resulting from active effort are more likely to benefit from individual therapy. (4.) Patients in extreme psychological distress may require a more supportive intervention, including hospitalization, before initiating psychoanalytic therapy. (5.) The degree and form of brain injury can affect the appropriateness of analytic treatment. Patients with significant expressive or receptive language deficits are not appropriate candidates. In addition to outlining the potential benefits of a psychodynamically oriented therapy for appropriately selected patients, Lewis and Rosenberg make two points that are generically applicable to the consideration of psychotherapy following traumatic brain injury. First, unaddressed psychological problems can be an impediment to meaningful participation in physical, cognitive and occupational rehabilitation, thus providing an additional argument for including psychotherapy in the treatment of some presentations of traumatic brain injury. Second, the patient's altered experience of self should not be viewed as an entirely neurological symptom. Brain injuries have psychological meaning.

"Although such disruptions (brain injury) can significantly affect the patient's self-esteem, and often represent a major focus for family work, they may represent a more basic and profound disturbance in the patients' sense of self. That is, beyond their difficulties in performing social roles, patients also struggle with the more fundamental question of who they are; the brain injury appears to disrupt severely their previously acquired self-image and sense of self [28]. Thus, a primary task of psychotherapy is to help the patient consolidate a new sense of self that successfully incorporates a realistic appraisal of strengths and weaknesses" [27].

In presentations where this alteration of sense of self is a significant element of the clinical presentation and the patient has sufficient ego development to tolerate an insight directed therapy, a psychodynamically informed therapy is indicated.

On reviewing psychotherapies appropriate for TBI patients, Folzer [29] made the following observation, "If 'immature' defenses and coping patterns are removed too early, the therapist may precipitate a catastrophe. Instead of directly confronting the patient, the therapist can introduce the focus on reality gradually." This would argue for a supportive therapy [30] instead of insight-oriented therapy. There is not, however, a strict division between these forms of therapy. As Werman [30] observed, "Although in the following pages these two forms of treatment (supportive therapy and insight-oriented therapy) are compared as if they were not only different from each other but virtually dichotomous in their aims and techniques, in reality they rarely exist in pure forms. Typically, over a period of time, most patients in supportive psychotherapy gain some insight into their behavior; similarly it is difficult to conceive of a course of insight-oriented psychotherapy in which some supportive measures are not utilized."

Psychotherapy following traumatic brain injury should not necessarily be limited to individual therapy. Several authors have emphasized the value of group therapy with TBI patients [29,31,32], and family involvement in therapy can be particularly important [12,23].

The discussion of psychotherapy with TBI patients and indeed psychotherapy in general has been conducted largely in the absence of quantitative data concerning the therapy itself. While standardized instruments for assessing baseline symptoms and treatment outcomes are increasingly being used in clinical research [33], these instruments do not quantify the fine structure of the therapeutic interaction. This contribution continues the development of quantitative methods for the characterization of patient-therapist communication with the long term objective of improving the effectiveness of psychotherapy following traumatic brain injury. Communication between patients and therapist during psychotherapy has many components including posture, eye contact, verbal tone, verbal production (the number of words exchanged irrespective of their meaning) and the manifest content of the communication. All of these interactions can be examined quantitatively [34,35]. For example non-verbal communication in the therapist-patient interaction has been analyzed by Yaynal-Reymond, et al. [36] and by Merten and Schwab [37] using a form of quantification developed by Magnusson [38,39]. While all components of patient-therapist communication are important, this paper focuses on content analysis. Using methods of symbolic dynamics this investigation extends previous analyses of the frequency of content by quantifying the temporally dependent, sequence-sensitive structure of the dialog. As long-term goals, the questions addressed in this research program follow those enumerated in Rapp, et al. [40].

1. Are there nonrandom patterns in the sequential structure of patient-therapist communication?

2. Do these patterns, should they exist, change during the course of therapy?

3. Do changes in the patterns of patient-therapist communication correlate with the clinically perceived success or failure of the therapy?

4. Can this type of analysis identify more effective forms of therapist intervention?

This case study is limited to an examination of the first three questions in three therapy sessions recorded from one patient. Generalized conclusions cannot therefore be made. The limited results do, however, indicate that there is a nonrandom structure in patient-therapist communication in these protocols. Additionally, quantifiable structures changed during the course of therapy in a manner that correlated with the clinically perceived success of the therapy.

### Quantitative investigations of patient-therapist communication: Prior Research

A first approach to quantitative content analysis is the determination of word frequency. An early effort was Electronic Verbal Analysis [41] measuring the frequency of anxiety related words. In a subsequent study, Pennebaker, et al. [42,43] recorded the frequency of 2800 words that were placed into seven categories, and Hart [44] analyzed political texts with a library of 10,000 words in five classes with approximately seven categories in each class. The limitations of these analyses are clear. Word frequency is insensitive to context. A randomly shuffled text will produced the same word counts. As Fast and Funder [45] observe, for example, the phrase "I am not happy" may be scored as positive emotional content.

Several investigators have developed methods that move beyond word frequency to examine meaning. A pioneer in this effort was Hartvig Dahl whose investigation of the case of Mrs. C analyzed 1,114 psychoanalytic sessions with the same patient [46-48]. In the 1974 study [48], entries in a three thousand word dictionary were assigned to one denotative category and to one or more connotative categories. A factor analysis was used to identify groups of related words, and it was shown that these groups were related to themes present in the transcript. In 1978 Dahl, et al. [49] published an application of linguistic analysis in psychotherapy that is intermediate to analysis of word count and the analysis of sequential structure based on symbolic dynamics presented in the next section. In this study, the analysis was limited to an examination of the therapist's interventions. This provides an instructive and valuable alternative to the practice of considering only the patient's speech. Each intervention by the therapist was classified by type and rated on scales designed to assess countertransference manifestations, including hostility, seductiveness, approval, disapproval and assertion of authority. A qualitative linguistic analysis based on Chomsky's model of transformational grammar [50,51] was also implemented. Dahl and his colleagues hypothesized that "a speaker has available a variety of syntactic options, and the particular syntactic structure which he chooses reflects, among other things, the inventory of wishes that he is motivated both to conceal and to express." The analysis of examples presented in this paper shows occasions of extraposition, pseudocleft construction, syntactic ambiguity and lexical ambiguity consistent with this hypothesis.

In the Gottschalk-Gleser analysis procedure [52,53], the grammatical clause is the unit of analysis. Content is scored on seven scales. In addition to the study of psychotherapy, Gottschalk-Gleser constant analysis has been applied in medical psychology [54-58]. GB Software markets a software product, PCAD2000, that applies a Gottschalk-Gleser content analysis to machine readable text. In addition to deriving scores on seven scales, the program offers a neuropsychiatric classification based on the DSM-IV.

Langs and colleagues [40,59] analyzed each element of therapy transcripts on fourteen dimensions. The result is a content matrix of fourteen columns. The analysis included calculations of the frequency of each entry, Shannon information of each column and the context free grammar complexity (Jiménez-Montaño, [60] described in the next section of this paper and in Appendix One). In the 1991 study [40], two one-hour protocols obtained from the same patient with different therapist were analyzed by this procedure. One therapist was a classically trained psychoanalyst. The other therapist used a communicative approach developed by Langs [61]. The most notable differences between the two protocols were the frequency of scores for the variable characterizing the sphere of reference (1 = therapy related, 2 = situations outside of therapy, 3 = reference to therapy and situations outside of therapy, 4 = unclear). In the case of the analyst, 90% of the material referred to situations outside of therapy and less than 1% referred to therapy related issues. In the case of the communicative therapist, 20% of the material focused on the therapeutic situation. Given the focus on the patient-therapist relationship in communicative psychotherapy, this observation is consistent with therapist expectations.

Stiles Verbal Mode Analysis [62-64] could be described as a statement classification method. The unit analyzed is an "utterance" (defined presently). Each unit is coded in to one of eight classes by a sequence of three forced-choice questions. Eight verbal response modes result. The analysis continues with a calculation of the frequency of each class. Verbal Mode Analysis is considered at greater length in the Discussion section of this paper.

Investigators have also examined the narrative speech of clinical populations using symbolic dynamics. In contrast with the research described above, these studies do not examine patient therapist communication. Rather they examine the sequence-sensitive structure of continuous narratives elicited by the question, "Can you tell me the story of your life?" [65,66] or a narrative produced by a participant in response to a request to recall the content of a story that they have just read [67].

The Leroy, et al. [67] study investigated the sequence-sensitive structure of a recall narrative presented by schizophrenic patients. Following Kintsch and Van Dijk [68,69], the participant's narrative was treated as a sequence of propositions. The Kintsch and Van Dijk definition of a proposition is the minimal semantic unit that can be either true or false. Propositions were classified as macro-propositions that specify the topic of discourse or micro-propositions that provide details. Macro-propositions were assigned the symbol "M," and micro-propositions were assigned the symbol "m." the narrative sample was thus recast as an ordered sequence of M's and m's. Entropy, Lempel-Ziv complexity and the first order transition matrix were calculated. Comparisons with surrogate data established the presence of a sequence-sensitive non-random structure in the data. The global complexity of recall did not differ for control and schizophrenic participants. There was, however, a difference in the transition structure. There were more micro-propositions to micro-proposition transitions in schizophrenic narratives.

In Doba, et al. [65] autobiographical speech of anorexics was parsed into 5 second epochs. Each epoch was assigned one of four symbols corresponding to negative emotion, positive emotion, neutral emotion and silence. In addition to distribution-determined measures, the Lempel-Ziv complexity and the first order transition matrix were examined. Complexity calculations with surrogate data established the presence of a non-random sequential structure in the narratives. In anorexics, dynamical measures identified recurrent cycles between expressions of negative emotion and silence that were less prominent in the control population. In a subsequent study [66], the same transcripts were analyzed with a different scoring system. Five symbols were used (family relations, social relations, love relations, self-reference and silence). Calculation of mutual information with the original symbol sequences and surrogate data sets again established the presence of a non-random dynamical structure in the narrative. The examination of the summed probability currents, a measure derived from the first order transition matrix, demonstrated that the narratives of anorexics are closer to statistical equilibrium than the narratives of controls.

## Methods

### Patient History

In this study, we describe the analysis of three therapy sessions (each separated by four months) conducted with the same patient (female, 32 years of age) by the same therapist (female). The patient had experienced several traumatic brain injures in a sequence of motor vehicle accidents two years prior to the initiation of therapy. The patient was referred by her psychiatrist for targeted psychotherapy treatment of pre-existing, non-suicidal self-injury and severe emotional dysregulation. Neurological examination established the absence of residual neurological deficits prior to the initiation of therapy. The accident history was, however, deemed to be psychologically significant and had a continuing negative impact on the patient's relationship with her partner. The patient received weekly individual outpatient therapy and group delivered training in behavioral skills. The analyzed sessions were from the individual therapy component. Each session was sixty minutes long. At the time of initiation of treatment the patient met DSM-IV diagnostic criteria for borderline personality disorder. This diagnosis was confirmed with a SCID-II (Structured Clinical Interview for Diagnosis) assessment. The patient was in dialectical behavior therapy following the methods developed by Linehan [70,71]. Treatment was ongoing between the sessions coded. Institutional Review Board and the participant's informed consent were obtained prior to initiation of the study. Therapy sessions were videotaped for subsequent analysis.

An assessment based on the DSM-IV was repeated at the end of treatment at which time the patient no longer met clinical criteria for a diagnosis of borderline personality disorder. Self report ratings of misery, depressed mood and anxiety were improved. Indices that brought the patient to treatment, frequent suicidal ideation and repeated self-injury, were no longer present and were not present at post-treatment follow-up six months after the termination of therapy.

### Restatement of the Protocol as a Symbol Sequence

There are several possible procedures for parsing a therapy protocol prior to restatement as a symbol sequence. One possibility is to set a fixed time interval and code the content of that interval. This was the procedure followed by Doba, et al. [65,66] who used 5 second intervals in their analysis of autobiographical speech. While having the advantage of explicit specification, this procedure has the disadvantage of being nonresponsive to the varying pace of natural dialog. We implemented here the more common practice, following Stiles [62-64,72] of parsing the protocol into natural speech elements. These elements are called utterances in the technical literature. As defined by Stiles, et al. [72] "The coding unit for both forms and intent is the utterance, defined as an independent clause, nonrestrictive dependent clause, multiple predicate, or term of acknowledgment, evaluation or address." A detailed presentation of the definition of an utterance which includes examples is given in Chapter 8 of Stiles' book "Describing Talk" [62].

Each unit of the protocol was assigned one or more symbols using the scoring system shown in Table 1. The protocol was thus reduced to a sequence of symbols drawn form a twenty-two symbol alphabet (Therapist: A, B, C, ... K, Patient: a, b, c, ...k) as shown in Table 1. This symbol set was chosen to emphasize elements that are prominent in a psychotherapy of borderline personality disorder based on dialectical behavior therapy [70,71]. Patient and therapist content was scored for all three sessions. In this preliminary case study parsing into utterances and symbol assignment was accomplished by the collective decision of three investigators. It is recognized that a more systematic investigation will require independent assessment and a quantitative test of inter-rater reliability. The following gives an example of each content type.

**Table 1:** Protocol Scoring Procedure

Therapist	Patient	Content
A	a	Acknowledging

B	b	Information (Requesting/Providing)

C	c	Request for Validation

D	d	Validating

E	e	Emotional Discharge

F	f	Complaint

G	g	Transitional/elicitation

H	h	Problem Presentation

I	i	Behavioral Analysis/Educational

J	j	Reflective

K	k	Irreverent

A symbol is assigned to specific content elements. Upper case symbols were used when the therapist was speaking, and lower case symbols were used when the patient was speaking.

Acknowledging: "Thank you for reminding me of that."

Information (requesting/providing): "I've had that car for two years."

Request for Validation: "Was I wrong to think that way?"

Validating: "Everyone feels that way from time to time."

Emotional Discharge "Never! Never! Never!"

Complaint: "My children never listen to me."

Transitional/Elicitation: "I wanted to remember to tell you about last Saturday."

Problem Presentation: "My husband lost his job."

Behavioral Analysis/Educational: "Do you think he would respond differently if you telephoned first?"

Reflective: "You seem to be saying that you wouldn't like that."

Irreverent "Well he certainly failed that time!"

Table 2. shows the distribution frequency of each symbol in the alphabet for all three sessions. The distribution computed using all sessions is unremarkable. The therapist's contributions consist primarily of acknowledgments, elicitations and problem presentations. The high frequency of patient complaints and emotional discharges is consistent with a diagnosis of borderline personality disorder.

**Table 2:** Symbol Frequency Distribution

Content	Symbol	Frequency All Sessions	First Session	Second Session	Third Session
P: Behavioral Analysis/Educational	i	.1184	.1073	.1475	.1010

P: Acknowledging	a	.0988	.0978	.0947	.1024

T: Acknowledging	A	.0899	.1041	.0638	.1038

P: Information (Requesting/Providing)	b	.0830	.0726	.0692	.0982

P: Complaint	f	.0798	.0915	.0692	.0827

T: Transitional/Elicitation	G	.0766	.0726	.0893	.0687

T: Problem Presentation	H	.0735	.0536	.0820	.0757

P: Emotional Discharge	e	.0697	.0820	.0820	.0547

T: Behavioral Analysis/Educational	I	.0659	.0820	.0729	.0533

T: Validating	D	.0532	.0915	.0474	.0407

P: Request for Validation	c	.0532	.0599	.0455	.0561

T: Reflective	J	.0450	.0221	.0492	.0519

P: Problem Presentation	h	.0317	.0221	.0346	.0337

T: Information (Requesting/Providing)	B	.0298	.0221	.0328	.0309

T: Irreverent	K	.0108	.0032	.0000	.0224

P: Transitional/Elicitation	g	.0108	.0032	.0182	.0084

P: Validating	d	.0051	.0126	.0018	.0042

P: Irreverent	k	.0044	.0000	.0000	.0098

T: Request for Validation	C	.0006	.0000	.0000	.0014

T: Emotional Discharge	E	.0000	.0000	.0000	.0000

T: Complaint	F	.0000	.0000	.0000	.0000

P: Reflective	j	.0000	.0000	.0000	.0000

The table shows the rank-ordered frequency of each symbol in the symbolic reduction. The expectation frequency is .0455. The letter T in the first column denotes therapist contributions and the letter P indicates patient contributions.

The symbol frequency distribution was also calculated for each session with a view to determining if longitudinal changes in symbol frequencies could offer insights into the patient-therapist interaction. We define a consistent change as one in which the frequency of appearance of a symbol either increases or decreases over all three sessions. In the case of the patient, only one variable showed a consistent pattern; the frequency of patient acknowledgments decreased. The decrease from Session 1 to Session 2 was, however, minimal. Otherwise, the only consistent patterns were seen in therapist behavior. The frequency of educational interventions decreased, and the frequency of reflective interventions increased. The frequency of validating interventions from the therapist decreased over the three sessions. This possibly reflects the growing confidence that both participants had in the therapeutic relationship.

Aside from describing predictable changes in therapist behavior, the analysis of symbol frequencies was largely uninformative. This is significant to the present investigation because it suggests the need for measures that quantify sequential behavior.

## Results

### Analysis of Repeated Pairs

The most elementary form of sequential analysis is the analysis of repeated pairs. The results from this analysis after combining all three therapy sessions are shown in Table 3. The expectation frequency of a repeated pair is p = .0021. Nine repeated pairs appear with a frequency that is at least one order of magnitude greater than the expectation frequency. Most of the repeated pairs are associated with what might be described as the mechanics of therapy: requesting, presenting and acknowledging information. As in the case of single symbol frequencies, patient complaints and emotional discharges appear frequently as elements in repeated pairs.

**Table 3:** High Frequency Repeated Pairs

First Element of Pair	Second Element of Pair	Frequency
T: Behavioral Analysis/Educational	P: Acknowledging	.0279

T: Problem Presentation	P: Acknowledging	.0260

P: Acknowledging	T: Behavioral analysis/Educational	.0247

P: Information (Requesting/Providing)	T: Acknowledging	.0241

T: Acknowledging	P: Information (Requesting/Providing)	.0234

T: Problem Presentation	P: Behavioral Analysis/Educational	.0234

P: Emotional Discharge	P: Complaint	.0228

T: Transitional/Elicitation	P: Behavioral Analysis/Educational	.0222

P: Complaint	T: Acknowledging	.0209

Most Frequently Observed Repeated Pairs. Analyzed over all three sessions, the frequencies of nine repeated pairs exceed the expectation frequency of .0021 by at least one order of magnitude. P denotes the patient, and T denotes the therapist.

### Analysis of Repeated Triples

When repeated triples are examined a marked pattern of change in content is seen over the three sessions. In a message of length L_M _there are L_M_-2 triples. Nonetheless there, are very few repeated triples in the clinical data. During Visit One nine triples appear more than 1% of the time. During Visit Two only two triples appear in more than 1% of the sample, and in Visit Three, four triples appear at a frequency exceeding 1% (Table 4).

**Table 4:** Repeated Triples Appearing at a Frequency Exceeding 1%

Visit One				
**Triple**	**Frequency**	**Content Symbol 1**	**Content Symbol 2**	**Content Symbol 3**

cef	.022	P: Request Validation	P: Emotional Discharge	P: Complaint

aIa	.019	P: Acknowledging	T: Behavioral Analysis/Educational	P: Acknowledging

IaI	.016	T: Behavioral Analysis/Educational	P: Acknowledging	T: Behavioral Analysis/Educational

bfA	.016	P: Information (Requesting/Providing)	P: Complaint	T: Acknowledging

efc	.013	P: Emotional Discharge	P: Complaint	P: Request for Validation

fAb	.013	P: Complaint	T: Acknowledging	P: Information

HaI	.013	T: Problem Presentation	P: Acknowledging	T: Behavioral Analysis/Educational

bAb	.013	P: Information (Requesting/Providing)	T: Acknowledging	P: Information (Requesting/Providing)

fce	.013	P: Complaint	P: Request Validation	P: Emotional Discharge

**Visit Two**				

Triple	Frequency	Content Symbol 1	Content Symbol 2	Content Symbol 3

aIa	.015	P: Acknowledging	T: Behavioral Analysis/Educational	P: Acknowledging

Iai	.011	T: Behavioral Analysis/Educational	P: Acknowledging	P: Behavioral Analysis/Educational

**Visit Three**				

Triple	Frequency	Content Symbol 1	Content Symbol 2	Content Symbol 3

bAb	.018	P: Information (Requesting/Providing)	T: Acknowledging	P: Information (Requesting/Providing)

IaI	.017	T: Behavioral Analysis/Educational	P: Acknowledging	T: Behavioral Analysis/Educational

AbA	.011	T: Acknowledging	P: Information (Requesting/Providing)	T: Acknowledging

aIa	.011	P: Acknowledging	T: Behavioral Analysis/Educational	P: Acknowledging

Repeated Triples Appearing at a Frequency Exceeding 1%. Results are presented separately for each session. P denotes the patient. T denotes the therapist.

There is a change in the content of repeated triples over the three sessions. In the first session the most frequently observed triple is a request for validation by the patient followed by an emotional discharge followed by a complaint. These three coding elements appear prominently in the other repeated triples observed during the first session. By the second session, which occurred four months after the first session, behavioral analysis by the therapist and acknowledgment of these communications by the patient are the most frequently observed triples. This pattern is consistent with clinical expectations. In the early sessions, the patient-therapist relationship is constructed by the therapist's nonjudgmental acceptance of the patient's complaints, emotional discharges and need for validation. This is particularly true in the course of borderline personality disorder. The work of therapy, implemented by behavioral analysis and education, begins after the construction of the therapeutic alliance.

### Context Free Grammar Complexity

While several methods can be used to characterize a symbol sequence, we consider first measures of complexity. Quantitative measures of complexity can be most readily introduced by considering an explicit example. Consider two messages, that is two symbol sequences, M_1 _and M_2_.

It should be noted that both messages have the same symbol frequency, eight appearances of each symbol. They are indistinguishable with distribution-determined measures, for example Shannon information, but M_2 _is more complex than M_1 _in our conventional understanding of the term. There are several methods for quantifying the complexity of symbol sequences. A taxonomy of complexity measures has been published [73]. In the first instance, we consider the context free grammar complexity introduced by Jiménez-Montaño [60] (a description is given in Appendix One). Consistent with our qualitative expectations, it is found that that grammar complexity of M_1 _is 20 bits and the complexity of M_2 _is 27 bits.

**Figure 1:**
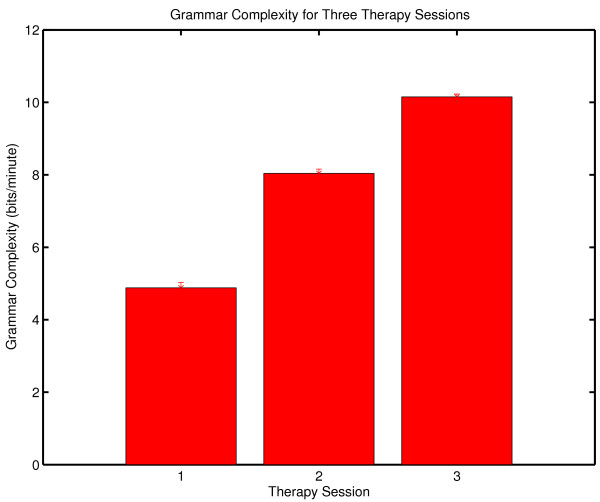
**Complexity generation in three psychotherapy sessions**. The context free grammar complexity of the symbolic reduction of each session was normalized against the duration of the session to determine complexity generation in bits/minute.

The complexity of an observed symbol sequence is often expressed in bits/unit time by dividing the complexity of the message by the period of observation [74]. The results from the three therapy sessions are shown in Figure 1. Complexity generation is seen to increase across the three sessions. (The procedure used to estimate the uncertainties of these complexity values is outlined in Appendix One).

This result is consistent with the increase in the number of symbols generated in the three sessions (N_DATA _= 317, 549, 713 respectively). While any observation based on a single case history must be stated circumspectly, the increase in the frequency of subject transition which is reflected in the increase in N_DATA _over the three sessions is consistent with qualitative clinical observations with borderline patients. As patients progress in therapy one can, in some instances, observe a decreased perseveration in topic and a greater flexibility of discourse. This result is consistent with the quantitative results of McDaniel, et al. [75] who found correlations between rate of improvement and an estimate of the number of patient utterances. The result seen here is also consistent with the Winefield, et al. [76] quantitative characterization of a psychodynamically oriented psychotherapy which showed decreasing asymmetry in patient/therapist verbal behavior during the course of treatment. This decrease in asymmetry was evidenced by increased therapist speech activity. Increased participation by the therapist would result in an increase in patient-to-therapist transitions in the symbol transcript, an increase in N_DATA_, and an increase in complexity generation.

**Figure 2:**
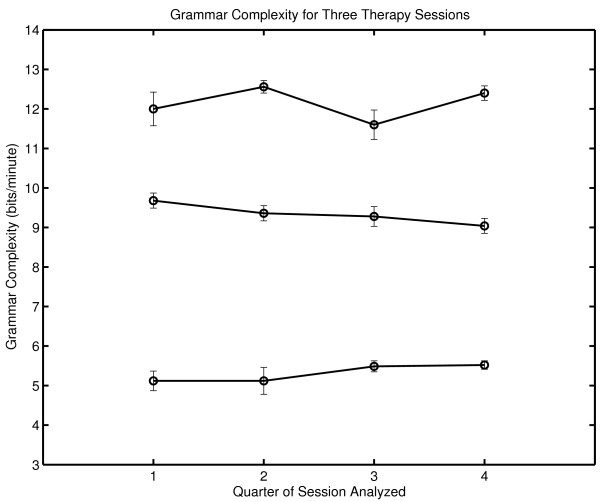
**Within session complexity generation for three therapy sessions**. Grammar complexity generation (bits/minute) was determined separately for each quarter of each session. The top curve corresponds to Session Three. The bottom curve corresponds to Session One.

It is also a matter of interest to determine the stability of complexity within a session. This was done by determining complexity generation for each quarter session. A visual inspection of Figure 2 suggests that there is a somewhat greater within-session variation in the third session. This is consistent with our understanding of an increase in complexity generation during the course of a successful therapy.

It is important to make a distinction between the complexity of a message and the intrinsic dynamical complexity of the system that generated the message. The intrinsic complexity of the generator can be estimated by comparing the complexity of the message against the complexity of random messages of equal length generated with the same symbol set. The result is the normalized complexity. Mathematical procedures for constructing this normalization are outlined in Appendix One. The normalized complexity is dimensionless and varies between a value close to zero for a constant symbol sequence (one symbol repeated throughout the entire message) and a value of one for a random sequence. Examples giving intermediate values of normalized complexity are shown in the appendix. The normalized grammar complexity of the three therapy sessions is .765 ± .033, .758 ± .015 and .763 ± .017. There is no significant change in the normalized grammar complexity which suggests that, at least in this therapy, grammar complexity did not detect changes in the underlying dynamical process. The contrast between the consistency of normalized complexity and the increase in complexity per unit time is considered in the Discussion section of this paper.

**Figure 3:**
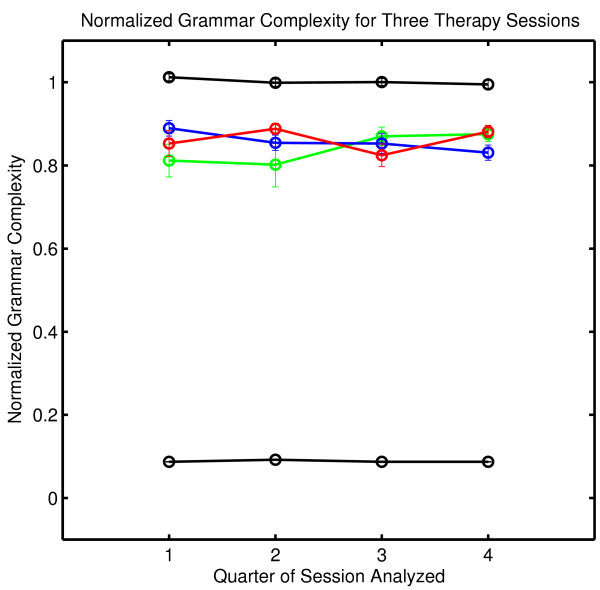
**Normalized grammar complexity for each quarter of each therapy session**. Normalized complexity is defined on [0,1]. The green line corresponds to Session One, the blue line to Session Two and the red line to Session Three. The complexity values obtained with random numbers (a black line at the top of the graph) and with a constant symbol sequence where one symbol is repeated throughout the message (a black line at the bottom of the graph) are shown for comparison. Data sets of the same size were used in the comparison calculations. The normalized complexity obtained with random numbers is approximately one, and the normalized complexity obtained with a constant signal is approximately zero. Details of the comparison calculations are given in Appendix One.

An examination of the normalized complexity for each quarter of a session allows an examination of the stationarity of the underlying dynamical process (Figure 3). The results are displayed on [0,1], the defined range of normalized complexity. There are no significant within-session or between-session differences when quarter sessions are analyzed.

A comparison of the complexity values obtained with the original therapy symbol sequence and complexity values obtained from random messages of the same length makes it possible to address the following null hypothesis:

As assessed by this complexity measure, the sequential structure of the original message is indistinguishable from the sequential structure of an equi-probable, random sequence of the same length constructed from the same symbol alphabet.

Several statistical tests of the null hypothesis have been considered (Appendix One). We use here the Monte Carlo probability of the null hypothesis.

N_SURR _is the number of comparison random messages (called surrogates) computed. The number of complexity values tested in the numerator includes the complexity of the original symbol sequence as well as the complexity values obtained with surrogates, ensuring that the numerator has a value of at least one. In the calculations in Figures 2 and 3, N_SURR _= 499 and C_Surrogate _> C_ORIG _in all cases. The null hypothesis is rejected with P_NULL _= .002; that is, the sequential structure of patient-therapist communication in these sessions as scored by this procedure and assessed by this metric is nonrandom.

### Lempel-Ziv Complexity

**Figure 4:**
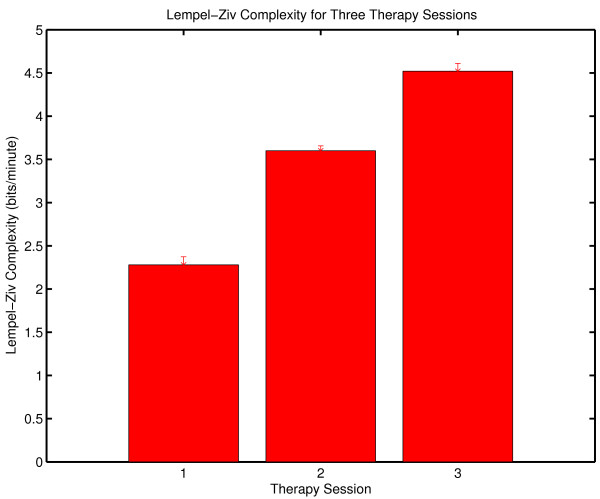
**Complexity generation in three psychotherapy sessions**. The Lempel-Ziv complexity of the symbolic reduction of each session was normalized against the duration of the session to determine complexity generation in bits/minute.

The results obtained with grammar complexity were confirmed by calculations of Lempel-Ziv complexity ([77] described in Appendix Two). Lempel-Ziv complexity and grammar complexity are in the same taxonomic group of complexity measures (randomness finding, nonprobabilistic, model based). The values obtained with Lempel-Ziv complexity are not the same as those obtained with the grammar complexity, but the two measures show the same sensitivity to randomness in a symbol sequence. The Lempel-Ziv results analogous to those obtained with grammar complexity are shown in Figure 4. As in the case of grammar complexity there is an increase in complexity generation over the three sessions.

**Figure 5:**
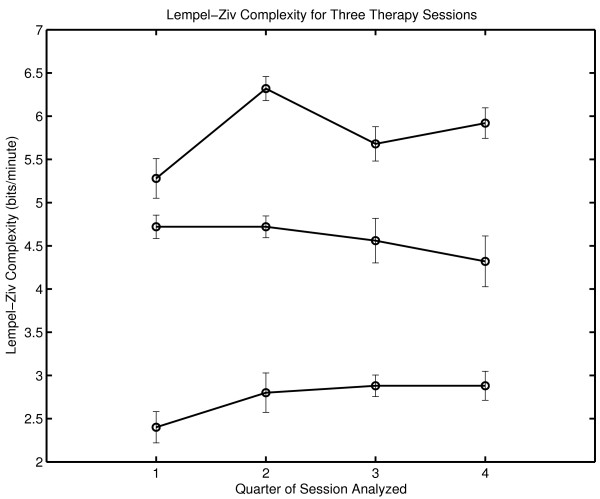
**Within session complexity generation for three therapy sessions**. Lempel-Ziv complexity generation (bits/minute) was determined separately for each quarter of each session. The top curve corresponds to Session Three. The bottom curve corresponds to Session One.

The within-session variability of Lempel-Ziv complexity (Figure 5) shows the same pattern that was observed with grammar complexity. The within-session variability is greater in Session Three.

**Figure 6:**
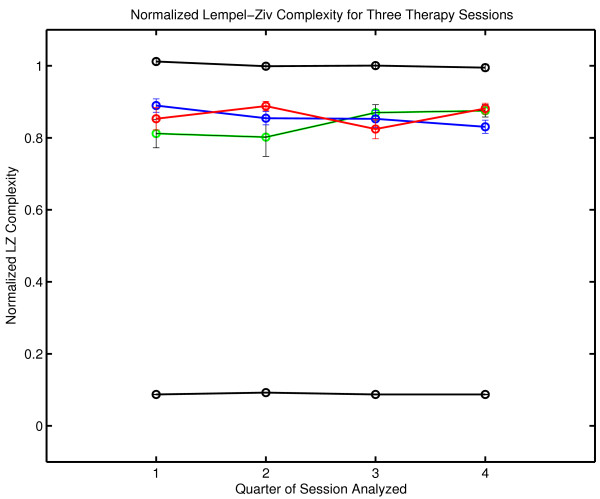
**Normalized Lempel-Ziv complexity for each quarter of each therapy session**. Normalized complexity is defined on [0,1]. The green line corresponds to Session One, the blue line to Session Two and the red line to Session Three. The complexity values obtained with random numbers (a black line at the top of the graph) and with a constant symbol sequence where one symbol is repeated throughout the message (a black line at the bottom of the graph) are shown for comparison. Data sets of the same size were used in the comparison calculations. The normalized complexity obtained with random numbers is approximately one, and the normalized complexity obtained with a constant signal is approximately zero. Details of the comparison calculations are given in Appendix One.

Lempel-Ziv complexity can also be normalized by comparisons with random surrogate symbol strings provided that the complexity of the surrogate is also determined with the Lempel-Ziv algorithm. The normalized Lempel-Ziv complexity for the three sessions is .765 ± .033, .758 ± .015 and .763 ± .017 respectively. In common with grammar complexity, no change in the generating dynamical process was detected with Lempel-Ziv complexity. These results should not be generalized inappropriately. It remains possible that significant change might be detected if a different measure was applied to the same data. It can only be said that normalized grammar complexity and normalized Lempel-Ziv complexity failed to detect any between-session changes while changes were seen in complexity generation rates with both measures. As previously noted, the between session consistency of normalized complexity and the increase in complexity per unit time is considered in the Discussion section. The within-session normalized complexity was also computed with the Lempel-Ziv algorithm (Figure 6). As in the case of grammar complexity, no significant within-session changes were seen in the normalized complexity.

As before, the surrogate null hypothesis of random structure was rejected by Lempel-Ziv complexity with P_NULL _= .002 (N_SURR _= 499) in all cases. It can again be concluded that patient-therapist communication has nonrandom structure.

### Mutual Information

Consider two simultaneously observed symbol sets Message A = (A_1_, A_2_, .....A_N_) and Message B = (B_1_, B_2_, .....B_N_) constructed from the same alphabet of N_α _elements. P_A_(I) is the probability of the appearance of Symbol I in Message A P_B_(J) is the probability of the appearance of Symbol J in Message B. P_AB_(I,J) is the probability that Symbol I appears in Message A and Symbol J appears in Message B at the same time. The average mutual information of Messages A and B is the average number of bits of Message B that can be predicted by measuring Message A. It is denoted by I(A,B). It can be shown [78] that

Mutual information is symmetrical I(A,B) = I(B,A). Also, if two processes are statistically independent then P_AB_(I,J) = P_A_(I)P_B_(J), and I(A,B) = 0. The special case where Message A and Message B are the same, I(A,A), is called self-information.

In this application, we examine the ability of a message to predict its own future. We define I(A_I_,A_I + 1_) as the mutual information observed when Message A = (A_1_,A_2_,.....A_N-1_) and Message B = (A_2_,A_3_,.....A_N_). This can be generalized to consider larger temporal displacements. I(A_I_,A_I + K_) is calculated by setting Message A = (A_1_,A_2_,.....A_N-K_) and Message B = (A_K_,A_K + 1_,.....A_N_). The time shifted self-information is a nonlinear measure of temporal decorrelation. Explanatory examples are given in Cellucci, et al [79]. If a message has strong temporal predictability then I(A_I_,A_I + K_) remains high as K is increased. If the process generating a message is dynamically disordered, then I(A_I_,A_I + K_) decreases rapidly as K increases.

Mutual information for the case K = 1 has been applied to the examination of the sequence-sensitive structure of narrative components in the autobiographical speech of anorexic adolescents and controls [66]. These investigators found that I(A_I_,A_I + 1_) is significantly lower in patients. They also compared I(A_I_,A_I + 1_) values obtained with their data against the values obtained with random shuffle surrogates and found that surrogates decorrelate faster than the original symbol sequence indicating the presence of non-random structure in the original symbol sequence.

**Figure 7:**
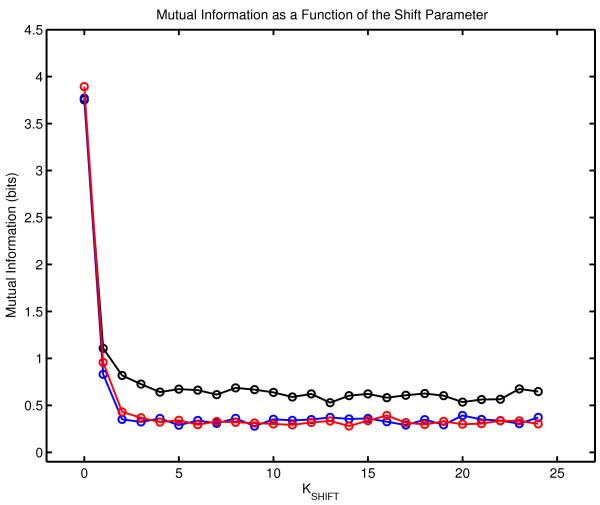
**Mutual Information as a Function of the Shift Parameter**. I(A_I_, A_I + K_) is shown as a function of K for the three therapy sessions. The black line corresponds to Session One, the blue line to Session Two and the red line to Session Three.

Figure 7 shows mutual information I(A_I_,A_I + K_) as a function of the temporal shift parameter K for the three therapy sessions. The mutual information measured in the first session decorrelates more slowly than mutual information obtained with Session Two and Three indicating a higher degree of predictability in Session One. This is consistent with the previous observation of a lower complexity generation rate in Session One. The separation of mutual information functions between the first and second session and the first and third session is significant (P < 10^-7^). This significance is computed by comparing twenty five values of mutual information (shift parameter K = 0 to 24) in a paired t-test. The mutual information values obtained in Sessions Two and Three are indistinguishable. This indicates that the process detected by longitudinal measurement of mutual information has stabilized by Session Two or that this measure is insufficiently sensitive to detect a continuing process altering patient-therapist communication.

The mutual information results obtained with higher values of the shift parameter K must be viewed with caution. A calculation of the mutual information of two symbol sequences tests their statistical independence. If the variables are independent, then P_AB_(i,j) = P_A_(i)P_B_(j) and I(A,B) = 0. It is possible to compute the probability of the null hypothesis of statistical independence. Let E_AB_(i,j) be the expected number of (i,j) symbol pairs given the assumption of statistical independence.

Let O_AB_(i,j) be the observed number of (i,j) symbol pairs. The corresponding value of χ^2 ^is

Where N_α _is the number of symbols in the alphabet. The number of degrees of freedom is ν = (N_α _- 1)^2^. The probability of the null hypothesis is

where Q is the incomplete gamma function.

When this analysis is applied to the symbol sequences generated by the three therapy sessions, the null hypothesis is rejected by construction for K = 0 but also for K = 1 for the three sessions. This result indicates the absence of predictive structures beyond the first symbol iteration, which is consistent with the results obtained with first order Markov surrogates in a later section of this paper.

### N^th^-Order Entropy and Conditional Entropy

The quantification of structure in symbol sequences with information theory begins with Shannon and the foundation of the subject [80]. Shannon subsequently developed procedures for investigating prediction and entropy in printed English ([81], extended by Burton and Lickliter [82], and by Cover and King [83]). Kolmogorov [84] considered the entropy of Russian texts in his seminal "Three approaches to the quantitative definition of information.". In this contribution we follow the development and notation of Ebeling and his colleagues [85,86]. Let  be the probability of the appearance of the i-th symbol in the alphabet in the symbol sequence being analyzed. We generalize this to consider the probability of each substring of length n, . We will use the term n-word to denote a substring of length n. The entropy of substrings of length n, denoted by H_n_, is given by

where N_max _is the number of possible n-words. N_max _will be a function of the size of the alphabet N_α_. In the absence of a priori rules restricting allowable n-words N_max _= (N_α_)^n^. The sum takes place over all substrings where . H_n _quantifies the average amount of information contained in a substring of length n, and therefore is monotone increasing in n. The related conditional entropies, h_n_, are given by

h_n _is the average amount of information needed to predict the next symbol in a substring if the first n symbols are known, giving h_n _≥ h_n + 1_.

**Figure 8:**
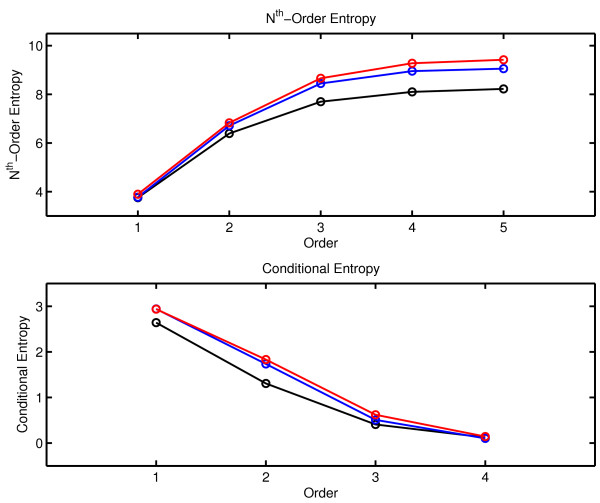
**N**^**th**^**-Order Entropy and Conditional Entropy for the three Therapy Sessions**. The black line corresponds to the first session, the blue to the second and the red line to the third session.

The values of H_n _and h_n _as a function of order n for the three therapy sessions are shown in Figure 8. At each order, the values of H_n _obtained in the third session are greater than the values obtained in the second session which are greater than the values obtained in the first session. This result is consistent with the previously presented rate of complexity generation (Session 3 > Session 2 > Session 1) and with the observation that mutual information, which is related to entropy, decorrelates faster in the later sessions. The between-session separation of conditional entropy is less marked, but the conditional entropy of Session 3 is greater than that of Session 1 at all orders of n.

As in the case of mutual information, the results of these entropy calculations must be viewed with care. A simple analysis indicates that length effects will cause a significant deterioration in an estimate of H_n _as n increases, if one uses the equation for H_n _given above. A message of N symbols contains N-(n-1) n-words. As previously noted the number of possible n-words in the absence of restrictive rules is (N_α_)^n^. Thus the number of possible n-words increases exponentially with order n, while the number of words actually present is limited by N. Let  be the expectation value of the number of appearances of the i-th n-word for the case of an equiprobable distribution.

The calculation of H_n _using the previous equation is warranted in the case of good statistics which is obtained when  is on the order of ten [87]. In the present analysis N_α _= 22, and the smallest value of N is obtained in Session 1 where N = 317. The criterion  is satisfied for n = 1 where H_n _for Session 1 < Session 2 < Session 3, but fails for n ≥ 2.

A further analysis shows that H_n _quickly approaches its limiting N-determined value as n increases. For a symbol sequence generated by the logistic equation near the Feigenbaum point, H_n _≈ log_2 _N, for large n where N is the length of the symbol sequence [88,89]. The same relationship is obtained with the computationally generated rabbit sequence [90]. These are highly disordered symbol sequences generated by deterministic processes. This argument can be generalized [87]. Recall that the number of possible words increases exponentially with n and is limited by N. To an approximation of the limiting case for large n, any given word is either absent or appears only once. In this case, there are N - (n - 1) ≈ N words with probability , and all others have . A series expansion can be used to show that . Therefore the limiting case of H_n _for large n is

In the case of the three therapy sessions, N = 317, 549 and 713 giving value of log_2 _N of 8.308, 9.101 and 9.478. The corresponding values of H_5 _are 8.225, 9.057 and 9.421. This suggests that the between-session differences in entropy as computed here simply reflects the increase in N over the three sessions.

Recognition of these issues has motivated the search for improved procedures for estimating H_n _when n is large and N is small. Several investigators have addressed this problem [88,91-97]. We have implemented on of these procedures [88] and applied it to the therapy data. As expected by the failure to satisfy the  criterion, no between-session separation was observed for higher values of n. These results are consistent with the conclusions of Lesne, et al. [97] who recommended using Lempel-Ziv complexity as the more reliable measure of structure when short symbol sequences are analyzed.

### Markov Surrogates

Let P_IJ _be the probability that Symbol I is followed by Symbol J. These probabilities are summarized in the first order transition matrix [P_IJ_]. A first order Markov surrogate is a symbol sequence constructed by a constrained randomization that has the same length and same [P_IJ_] as the original symbol sequence. A comparison of complexity values obtained with Markov surrogates and the complexity of the original symbol sequence can be used to address the following null hypothesis:

As assessed by this measure, the sequential structure of the original message is indistinguishable from the sequential structure of a random process that has the same length and first order transition matrix as the original message.

Calculations with Markov surrogates follow the same pattern as calculations with equi-probable surrogates. C_ORIG _is determined, surrogates are constructed (in this case first order Markov surrogates), and values of C_Surrogate _are calculated. The probability of the null hypothesis is calculated using the previous formula and these values of C_ORIG _and C_Surrogate_. With these data and these complexity measures, there is a failure to reject the null hypothesis for all three sessions. The average value of P_NULL_obtained with the context free grammar complexity and 499 equi-probable surrogates was .935 and the average value obtained with Lempel-Ziv complexity was .617. This means that with these data and these measures of complexity, a therapy session's symbol sequence is indistinguishable from a random process with the same first order transition matrix. This does not mean that a higher order structure is not present in the sequence. Rather, the results show that these measures failed to find evidence for that structure. Theoretically, the null hypothesis could be rejected with these data and a different measure.

## Discussion

This is a case study, and therefore any results must be regarded as inconclusive until confirmed by a more systematic investigation. In this therapy the rate of complexity generation increased across the three sessions investigated. This increase in variability is consistent with the statistically significant faster decorrelation time observed in the K = 1 mutual information calculation and in the increase in n-th order entropy and conditional entropy for n = 1. It is also consistent with the clinically observed changes in the flexibility of patient communication during the course of treatment. Additionally, using two measures of complexity we have demonstrated that the sequential structure of patient-therapist dialog in these sessions has a nonrandom structure (P_NULL _= .002). These results are consistent with results of previous investigations summarized by Leroy, et al. [57]:

"(1) temporal organization is a significant feature of speech,

(2) counting (by which they mean the sequence-independent, distribution-determined frequencies of content elements) is not sufficient for an adequate characterization of language, and

(3) symbolic dynamical methods are needed for the sake of completeness"

As previously noted, the contrast between the consistency of normalized complexity (both Lempel-Ziv and context free grammar complexity) over the three sessions and the increase in complexity generation (complexity per unit time) requires examination. Possible insights into this question can be gained by examining the quantitative literature investigating hierarchical structures in language [98-101]. Based on this research we wish to suggest that the normalized complexity quantifies an invariant structure intrinsic to language when characterized by this form of symbolic restatement, while complexity per unit time quantifies pragmatic language use. Some measure of support for this hypothesis can be obtained by consideration of work by Montemurro and Zanette [102]. Montemurro and Zanette examined the sequential structure of word ordering in 7,097 texts drawn from eight languages (English, French, German, Finnish, Tagalog, Summarian, Old Egyptian and Chinese). They computed a measure of entropy based on Lempel-Ziv complexity and a normalized relative entropy based on comparisons with randomly shuffled sequences of equal length. They found that "while a direct estimation of the overall entropy of language yielded values that varied for the different families considered, the relative entropy quantifying word ordering presented an almost constant value for all these families. ... Therefore our evidence suggests that quantitative effects of word order correlations on the entropy of language emerges as a universal statistical feature." The Montemurro and Zanette study examined the sequential structure of word ordering. It is recognized that word ordering is not identical to the concept sequencing uncovered by the symbolic coding process used in our study, but the presence of near constant normalized complexity in the presence of a highly variable complexity offers support, albeit indirect support, for our hypothesis of a dissociation between pragmatic complexity (bits/minute) and intrinsic structure (normalized complexity). This is a case study of a single patient. Any further speculation must be deferred until additional data are available.

A systematic research effort will be required to address the other questions raised in the introduction to this paper. In addition to acquisition of longitudinal data obtained from a large, clinically homogeneous population, the introduction of additional measures of sequential structure can be considered. The inverted-U measures of complexity [103] are theoretically important but have received little application with observational data (as distinct from computationally generated symbol sequences). These measures of complexity give low values for both highly regular sequences and for random sequences but high values for chaotic sequences (where the word chaos is being used here in the technical sense). This is an interesting possibility since it has been suggested that patient-therapist communication can be chaotic [104-109]. More general reviews of dynamical metaphors in psychopathology and psychotherapy are given in [110-113].

An analysis of the computational results identified the limitations of the approach taken in this paper. The large number of symbols in the alphabet and the comparatively short message lengths severely limit the kinds of analyses that can be applied. An alternative approach can be implemented using Stiles' Verbal Mode Analysis. In this analysis, each utterance is scored by three forced choice questions called principles of classification (source of experience, frame of reference, and presumption). These three binary scores are used to specify one of eight mutually exclusive categories. The eight celled classification process is applied to each utterance twice. The first classification is determined by grammatical form. The second is based on pragmatic intent. Several analysis options are thus available. The sequential structure of the form (grammatical) coding and the intent (pragmatic) coding can be analyzed separately. In these cases, N_α _= 8. At a finer scale, the three principles of classification each generate a binary sequence, N_α _= 2 that can be examined. These scoring methodologies make it possible to perform mathematical analyses that are not feasible for large N_α_. The analyses of ordered triples reported here does, however, indicate that the rich content introduced by a large N_α _can reveal important insights into the evolving dynamic of patient-therapist communication. In the ideal case, protocols can be scored by more than one procedure and analyses performed with the widest possible collection of mathematical methods.

Psychotherapy, even when the treatment is concretized in treatment manuals is, by nature, transactional, flexible and often highly individualized. As such, the field of psychotherapy research standardly employs a 'technology model' [114] in conducting treatment development and evaluation research. Psychotherapy process researchers also employ a methodology for measuring complex, deterministic, and dynamic processes within the therapy experience. Core to both models is the application of highly specified behavioral coding systems to recoded samples of therapy sessions. A discussion of this literature is beyond the scope of the current article. Also, it should be noted that our experience suggests that the procedures presented here are not only generically applicable to the field of psychotherapy research but are also of value in training therapists. The discipline of examining each verbal exchange at this level of detail and the process of identifying recurring patterns of communication (the words change, but the symbol sequence recurs) helps trainees to identify maladaptive communication strategies and encourages them to view a therapy not as separated exchanges but as a larger scale dynamical process. Independently of the subsequent mathematical analysis, the process of sequential symbolic transcription is a valuable exercise. Additionally, these methods may be of particular value in the examination of therapies following traumatic brain injury. These therapies can, in some instances, be complicated by cognitive deficits that result in distortions of language. As noted by Granacher [115] a distinction is to be made in the language deficits following traumatic brain injury between deficits of speech (the mechanical articulation of language) and deficits in the use of language (generation and comprehension of syntactic and semantic structure) which can be investigated using the methods tested here. In the case of injuries, frank aphasias can result. Their identification does not require sophisticated mathematical analysis. These aphasias typically resolve spontaneously into mild residual anomia [116,117]. In other cases, however, subtle distortions of language can occur after traumatic brain injury. "The basic structural components of language may be intact but the ability to use language to engage socially is impaired." [117] Deficits in the effective use of language following traumatic brain injury have been reviewed by Coelho [118] and by Coelho and Youse [119]. In addition to complicating therapy, these deficits can have a significant negative impact on post-injury quality of life. These deficiencies in language are commonly described as deficits in pragmatic competence where, as used here, the word pragmatics is defined as the subfield of linguistics which investigates the way in which context contributes to meaning [120,121]. These deficits are not typically expressed as failures to comprehend single sentences but are observed as failures to understand sequence-dependent, multi-sentence discourse [122]. Sohlberg and Mateer [117] have provided the following summary:

"Pragmatics constitute a comprehensive set of skills required for competence in naturalistic, functional use of language. The term can be broadly defined as the use of language for communication in specific contexts [123]. Pragmatics behaviors transcend isolated word and grammatical structures; they make up the system of rules clarifying the use of language in terms of situational or social contexts. People with brain injury often demonstrate normal basic linguistic skills, but have difficult adapting their communication to specific contexts; for example they may exhibit tangential speech, poor verbal organization, or inadequate turn taking [124]."

Pragmatic deficits are not limited to traumatic brain injury but are also observed in autism [125,126], attention deficit hyperactivity disorder [127] and schizophrenia [128,129]. In contrast with these disorders, the presentation of deficits in pragmatic competence following traumatic brain injury is complicated by acute onset followed by a complicated post-injury time course that can result from progressive cognitive loss or improvement due to spontaneous resolution. Highly variable day to day changes in competence can also sometimes be observed.

As reviewed by Martin and McDonald [121], three theories presenting explanations for deficits in pragmatic competence following traumatic brain injury are now under consideration: Social Inference Theory, Weak Central Coherence and Executive Dysfunction. Social Inference Theory argues that pragmatic failures follow from failures of the patient's Theory of Mind. An individual's Theory of Mind is "the capacity to infer mental states of others ... a person's ability to form representations of other people's mental states and to use the representations to understand, predict and judge utterances and behaviors" [130]. Following initial work by Santoro and Spiers [131], a rapidly growing literature has documented Theory of Mind deficits following traumatic brain injury [132-136]. Weak Central Coherence results in an individual's focus on components and a failure to integrate components into larger scale coherent structures. In addition to being a possible cause of post-injury pragmatic deficits, weak central coherence may be present in autistic patients [137-139]. The Executive Function system controls and regulates other processes and is particularly important in responding to novel situations requiring planning and decision making. Executive functions are localized in the prefrontal cortex [140,141], and injury to the prefrontal cortex can cause executive dysfunction which in turn results in deficits in language. Significant correlations between executive function and pragmatic communication difficulties following traumatic brain injury have been reported [142].

While arguments can be made that deficits in any of these capabilities (Theory of Mind, Central Coherence, and Executive Function) can result in pragmatic language deficits, there is no present evidence indicating which of the three is predominant in pragmatic deficits following traumatic brain injury. Indeed, several investigators have results indicating that it is very difficult to ascribe observed deficits to any given cause [143-146]. Given the heterogeneity of the traumatic brain injury population, it seems probable that pragmatic failures will have different causes in different patients.

Irrespective of the cause, psychotherapists of traumatic brain injury patients must be sensitive to the possible impact of erratically varying language competence in patient-therapist communication. As outlined in Sohlberg and Mateer [117] none of the currently available procedures for assessing pragmatics following brain injury are completely satisfactory. The methods closest to the procedures developed here are conversational analysis studies of language following traumatic brain injury [147-151]. The analysis employed by Snow, et al. [147] was modified from Damico's Clinical Discourse Method [152,153]. Seventeen parameters were organized into four groups (Quantity, Quality, Relation, and Manner). A similar study published by Friedland and Miller [149] scored natural conversations in four areas (Repair, Silences, Minimal Turns, Topic). In a study of conversational structure, Coelho, et al. [151] found differences in the flow of conversation of traumatic brain injury patients when compared to healthy controls. They found that patients were more dependent on the investigator to maintain the interaction. Individuals with traumatic brain injury did not initiate and appeared to function primarily as responders. The sequential structure of discourse is not, however, quantified by these methods. It can be noted, however, that it may be possible to apply the sequence sensitive measures presented here to these previous analyses. For example, in the Coelho, et al. [151], study two categories of analysis were applied, Appropriateness and Topic Initiation. The Appropriateness of an utterance was assigned to one of four categories (Obliges, Comments, Adequate Responses, Adequate Plus Responses). The results were reported as between-group means and standard deviations. Significant differences were seen in two of the four categories. This analysis can be viewed as a restatement of the conversation as an eight-symbol alphabet (four Appropriateness categories crossed against Investigator or Patient). The sequential structure of this symbol sequence can be quantified.

How might deficits in pragmatic competence following traumatic brain injury be reflected in complexity and entropy measurements of patient-therapist communication? Deficits in pragmatics are reflected in sequential structures including poor organization, tangential speech and loss of coherence. As documented by Chapman [154,155] some patients will present a loss, possibly a subtle loss, of coherence in verbal production. This loss of coherent structure would be reflected in an increase in complexity and entropy, a more uniform symbol frequency distribution and a broader spectrum of repeated pairs. Conversely, perseverations of discourse and topic repetitiveness, which can also be observed following brain injury [156], would result in a decrease in complexity and entropy. Pragmatics deficits would therefore be expected to produce bimodally distributed values of sequence sensitive measures. Further research may show that a high degree of variability in complexity within sessions and between sessions is diagnostic of failures of pragmatic competence in traumatic brain injury patients.

The present results suggest that dynamical measures can be used longitudinally to follow the course of treatment. To a degree, it is possible to consider two distinct processes occurring during the course of psychotherapy following a brain injury: psychological change reflecting emotional development and cognitive change having an impact on language due to organic changes in the central nervous system (recovery or continuing deterioration). The longitudinal application of the measures developed here may provide a means of separating and observing these processes quantitatively. Psychological change may be reflected in changes in subject content while cognitive changes may result in changes of linguistic structures that can be captured by complexity measures.

## Conclusions

We join with Morris and Bleiberg [157] in arguing that psychotherapy should be integrated with cognitive rehabilitation in the treatment of brain injury patients. We also agree with Judd and Wilson [158] in concluding that modifications of psychotherapy will often be required when working with these patients. The diversity of the traumatic brain injury population makes it impossible to construct a single, generic therapy for these patients. Any conclusions based on the quantitative analyses of protocols from a single patient are clearly provisional. It is suggested, however, that with further development and larger studies, the methods developed here for the quantitative analysis of dynamical structures in patient-therapist communication may become useful on a patient-by-patient basis to inform these clinical decisions.

## Competing interests

The authors declare that they have no competing interests.

## Authors' contributions

PER wrote the software to compute Lempel-Ziv complexity, software to confirm calculations of context free complexity, performed the complexity calculations and was the primary author of the manuscript. AMKG supervised the statistical analysis of the results. MAJM wrote the software for grammar complexity calculations and computed the repeated pairs analysis. CJC contributed calculations of mutual information, n-th order entropies and conditional entropies. KEK was the treating psychotherapist and led the process of restating the therapy protocols as symbol sequences. All authors have read and approved the final manuscript.

## Appendix One. Context Free Grammar Complexity

Classically complexity is defined as the amount of information required to specify the contents of a message [84,159,160]. An historical review is given in Li and Vitányi [[161] Section 1.6]. This definition can be operationalized by building an instruction set that can generate the message. The complexity of the message is defined to be the length of the instruction set. This operationalization is implemented in the context free grammar complexity [60,162]. A systematic procedure (outlined below) is used to construct an algorithm that can reconstruct the original message. The size of the algorithm (also defined below) is the complexity of the message. It is understood that this gives an upper bound to complexity. It is always possible that an alternative construction will give a smaller instruction set. This is true of all complexity measures in this category (randomness finding, nonprobabilistic, model based, [73]). Because the procedure used to construct the algorithm is systematic, complexity is valid as a comparative measure. This consideration also indicates why it is useful to have the results of a complexity analysis confirmed by the application of a second measure. "There is no single value of complexity. These calculations provide a systematic procedure for obtaining an empirical measure of dynamical behavior that can be compared across conditions." [163].

The procedure for determining the context free grammar complexity is best introduced by considering a specific example. Consider the previously introduced message M_2_.

The procedure begins with a search for repeated pairs. In this message, the pair AD is the most repeated pair. It is replaced by the new symbol α = AD.

BC is the next most frequently repeated pair. It is replaced by symbol β = BC.

BB is repeated twice, but as will be seen replacing a pair of symbols with a new symbol does not result in a decrease in the size of the instruction set if the pair is only repeated twice. The search for repeated pairs therefore ends, and the search for repeated triples begins. The triple BBD is repeated twice. In the case of triples, replacing a repeated triple does decrease the size of the instruction set even if it is only repeated twice. BBD is replaced by γ

There are no other repeated triples. In the general case, the search for repeated triples is following by a sequential search for repeated n-tuples, n = 4, 5, 6.... until the search is exhausted. In the case of this message there are no higher order repeats. The compression has converged.

In the next step product terms are replaced by exponentials. Thus AA is replaced by A^2^. CCC is replaced by C^3 ^and BB is replaced by B^2^. The instruction set to reconstruct the original message is:

Complexity is determined by calculating the size of this instruction set. Under the definition used here [60] each symbol adds one to the complexity and exponents contribute logarithmically (base 2).

The total is 27.585. Again under this definition, the integer part of the final sum is reported in bits. The context free grammar complexity of M_2 _is 27 bits.

Estimating the uncertainty in the complexity of a specific message is problematic when the message is considered in isolation and a large population of messages generated by the identical dynamical process is not available. In Rapp, et al. [164] the uncertainty in C_ORIG _is approximated by finding the difference in complexity values obtained in the first half and the second half of the message and expressing this difference as a fraction of their average value. Let C_A _be the complexity of the first half of the message. Let C_B _be the complexity of the second half of the message. Under this approximation, the uncertainty in C_ORIG _is given by

where we use the property C_A _and C_B _are positive.

This procedure can give an aberrant value of zero when C_A _= C_B_. An alternative procedure for estimating ΔC_ORIG _can be constructed by calculating <C_1/2_>, the mean value of complexity calculated from all possible substrings of length L_M_/2, and σ_1/2 _the standard deviation of that mean. Expressed as a fraction, uncertainty is σ_1/2_/<C_1/2_>, and ΔC_ORIG _is given by

This procedure is, however, computationally insupportable for longer messages. Suppose L_M _= 8000. This procedure for estimating <C_1/2_> would require averaging 4000 values of complexity calculated from strings of length L_M_/2 = 4000. We have adopted the procedure of calculating <C_1/2_> from 100 strings of length L_M_/2. They are selected randomly from the set of all possible L_M_/2 substrings. In cases where L_M _< 200, <C_1/2_> is calculated from all possible substrings of length L_M_/2.

**Figure 9:**
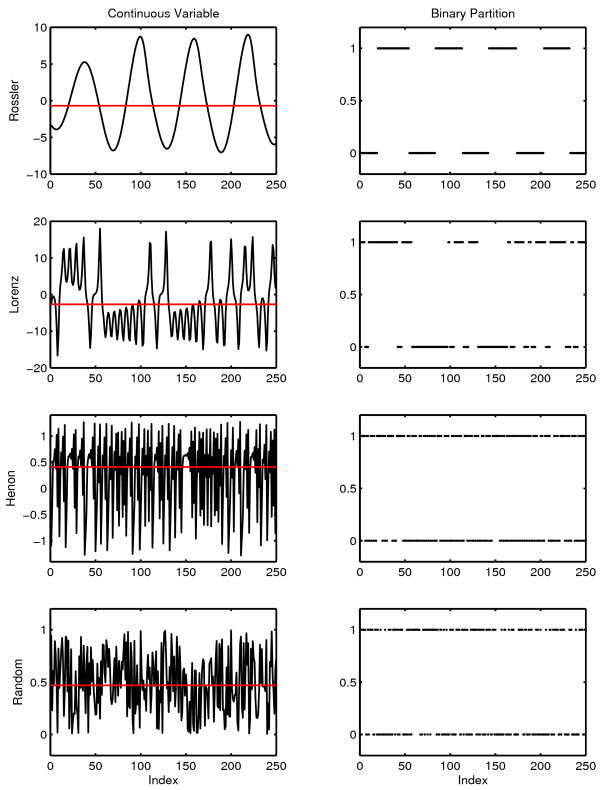
**Partitioning real data onto a discrete symbol set**. The median is determined form the original data. In this example, real variables are replaced by the symbol '0' if they are less than the median and by symbol '1' if they are greater than or equal to the median. The locations of medians are indicated by the horizontal red lines. Graphs in the left column show the real variable time series. The corresponding symbol sequences are shown in the right column.

A qualitative understanding of the complexity of a symbols sequence can be obtained by applying these measures to symbol sequences generated by standard systems that are commonly examined in dynamical systems theory. Five examples are considered here: a constant sequence (the same symbol is repeated), sequences generated by the Rössler and Lorenz systems (both three dimensional ordinary differential equations), the Hénon system (a two dimensional difference equation) and a random number generator. The technical specifications of the systems are given in Appendix Three. The Rössler, Lorenz, Hénon and random data are expressed as real variables. In order to apply a symbolic dynamics-based measure of complexity, it is necessary to project these data sets to a discrete symbol set. There are several possible procedures for doing this. Radhakrishnan, et al. [165] used K-means clustering. While conceptually attractive, the results of K-means clustering can be very sensitive to initial conditions. Bradley and Fayyad [166] addressed this sensitivity by constructing a K-means algorithm that produces a refined initial condition that improved performance. Insofar as we know, this method has not been applied to the problem of converting real data to symbolic data. An alternative approach has been published by Hirata, et al. [167] who approximate a generating partition from a time series using tessellations. This is a computationally demanding procedure and there are practical issues concerning the sensitivity of the partition on the initialization. Steuer, et al [86] recommend using the partition that maximizes entropy. In the present examples, the continuous variable time series is partitioned about the median. In this process, the median is computed from the original time series. A real variable is replaced by symbol '0' if it is less than the median and by symbol '1' if it is greater than or equal to the median. The choice of the median rather than the mean is critical to this process. False-positive indications of deterministic structure in random data can result if the mean is used [168]. The partitioning process is depicted in Figure 9. (It should be noted that in the present paper, the consideration of partitioning protocol only applies to the didactic examples presented in the appendices. The psychotherapy data are symbolic and partitioning is not required).

**Figure 10:**
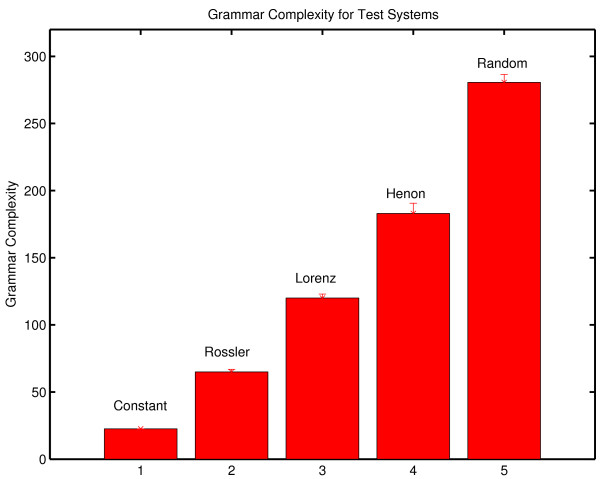
**Grammar complexity for one thousand element symbol sequences generated by the model systems**. Symbol sequences were generated by the partitioning procedure outlined in Figure 9.

The grammar complexity values computed from one thousand element symbol sequences generated by these model systems are shown in Figure 10. The results are seen to be consistent with our qualitative understanding of complexity. The constant sequence gives the lowest value, and the random number generator produces the largest value. The ordering Rössler less than Lorenz, less than Hénon is also consistent with expectations based on a visual examination of the time series in the left column of Figure 9.

**Figure 11:**
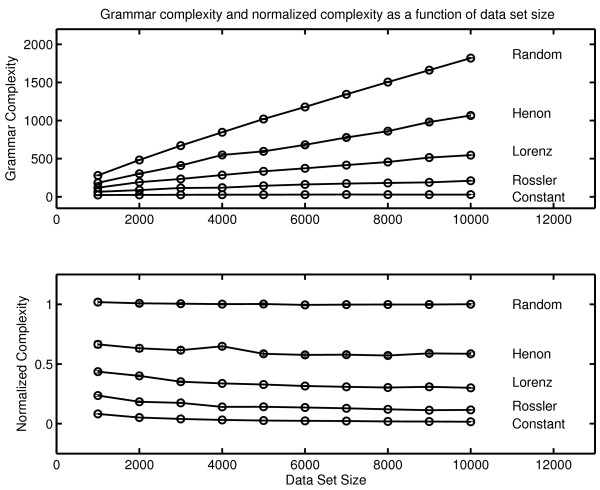
**Grammar complexity and normalized complexity as a function of data set size**. Symbol sequences were generated from the previously defined model systems using a binary partition about the median. The upper panel shows the grammar complexity. The lower panel shows the corresponding normalized complexity where values of grammar complexity are normalized against random, equi-probable surrogates. In these calculations, 499 surrogates were used to compute C_N_.

A critical distinction must be made between the complexity of a message, C_ORIG _and the intrinsic complexity of the process that generated the message. The value of grammar complexity will depend on two factors, the complexity of the dynamical process generating the symbol sequence and the length of the symbol sequence. This is seen in the upper panel of Figure 11 where grammar complexity is plotted as a function of the length of the data set. The ordering of complexity values seen with 1000 element sequences in Figure 2 is preserved (random > Hénon > Lorenz > Rössler > constant) and the values increase with the size of the data set. It is therefore necessary to find an effective normalization of complexity values that allows comparison of intrinsic complexities without the complication of data set size.

It might be supposed that dividing C_ORIG _by the length of the message is an acceptable solution. It has been shown that this is not the case [169]. An effective normalization of C_ORIG _can be achieved by comparing it against the values of complexity obtained from random equiprobable messages of the same length. Let N_α _be the size of the symbol alphabet (the number of distinct symbols available for message construction, in these examples N_α _= 2). N_α _is not message length L_M_. An equiprobable surrogate is one where each symbol appears with probability 1/N_α_. Let <C_S_> denote the average value of complexity obtained from random equiprobable surrogates of length L_M _(the subscript s denotes a surrogate). The normalized complexity is defined by

C_N _ranges from close to zero for messages containing a single repeated symbol to close to one for messages generated by random processes.

As outlined in Rapp [170], C_N _cannot be formed by normalizing against random shuffle surrogates. Consider the case of a message that consists of a single repeated symbol selected from an alphabet of size N_α _> 1. (Sequences in a message space of N_α _= 1 consist of a single symbol and only differ by length. Trivially, their complexity is the number of bits required to encode length L_M_.) An effective normalization should give a low value of C_N _to a repeated symbol message. Suppose surrogates were formed by a random shuffle. Since the message contains only one symbol, they all have the same value of complexity. In this case, C_ORIG _= <C_S_> and hence C_N _= 1, which is the complexity of a random message. If instead surrogates are equiprobable on N_α_, N_α _> 1, then <C_S_> is greater than C_ORIG _and C_N _has a low value. A low value of complexity is expected for a constant sequence.

The uncertainty in C_N_, ΔC_N_, can be estimated by the following argument

The estimation of ΔC_ORIG _has been discussed. <C_S_> is the mean complexity computed from a distribution of equiprobable surrogates. Δ<C_S_> is the standard deviation of that distribution.

Comparison of C_ORIG _and the complexity values obtained with surrogates makes it possible to address the following surrogate null hypothesis:

As assessed by this complexity measure, the sequential structure of the original message is indistinguishable from the sequential structure of an equi-probable, random sequence of the same length constructed from the same symbol alphabet.

Several statistical tests of the null hypothesis have been considered [168]. We use here the Monte Carlo probability of the null hypothesis.

N_SURR _is the number of surrogates computed. The number of complexity values tested in the numerator includes the complexity of the original symbol sequence as well as the complexity values obtained with surrogates, ensuring that the numerator has a value of at least one. This statistical test was chosen because it is a distribution-agnostic test, that is, it makes no assumptions about the structure of the C_Surrogate _distribution. Surrogates have a random structure, and the grammar complexity gives the highest value to random sequences. We therefore expect the values of C_Surrogate _to be greater than the value of C_ORIG _if a nonrandom structure is present in the original sequence. The smallest value of P_NULL _will be obtained when all values of C_Surrogate _are greater than C_ORIG_. That minimum value is therefore 1/(1 + N_SURR_). In the calculations in Figures 10 and 11, N_SURR _= 499 and C_Surrogate _> C_ORIG _in all cases for the constant sequence, Rössler, Lorenz and Hénon data. The null hypothesis is therefore rejected with P_NULL _= .002. As expected, the null hypothesis is not rejected by symbol sequences produced by a random number generator. For the ten cases in Figure 11 corresponding to L_M _= 1000, 2000, ... 10,000, the average value of P_NULL _obtained with random data is P_NULL _= .562

Barnard [171] and Hope [172] have proposed a nonparametric test for rejecting the null hypothesis. Under their criterion the null hypothesis is rejected if C_ORIG _< C_Surrogate _for all of the surrogates. If this criterion is met, as it is in these calculations, the probability of the null hypothesis is again P_NULL _= 1/(1 + N_SURR_).

As outlined in Watanabe, et al. [163] reported values of complexity obtained with real variable data requires the specification of:

1. the complexity measure used,

2. the number of symbols in the alphabet,

3. the procedure used to partition values onto the symbol set,

4. the procedure used to generate the surrogates used to calculate C_N_,

5. the number of surrogates used, and

6. the statistical procedure used to calculate the probability of the surrogate null hypothesis.

## Appendix Two. Lempel-Ziv Complexity

As before let message M be a finite symbol sequence of length L_M_. The vocabulary of a symbol sequence, denoted by v{M}, is the set of distinct subsequences that can be found in the message. By definition, a message is an element of its own vocabulary. If, for example, M = 00101, then:

A message can be expressed as a concatenation of substrings. Thus M = 000110100 is equivalent to M = X_1_X_2_X_3_X_4_, where X_1 _= 0, X_2 _= 001, X_3 _= 10, and X_4 _= 100. An additional element of notation is required. For any message M, the message MM_π _is the identical message following deletion of the last symbol. M_π _therefore has length L_M_-1. This deletion operation can be combined with concatenation. If X_1 _= 001 and X_2 _= 011, then (X_1_X_2_)_π _= 00101.

For any symbol sequence of length L_M _≥ 3, more than one decomposition into substrings is possible. The Lempel-Ziv algorithm [77] prescribes a procedure for decomposing a message into a concatenation of substrings. Only one decomposition is consistent with the algorithm. The Lempel-Ziv complexity is defined as the number of subsequences produced by this decomposition. In a Lempel-Ziv decomposition, the first subsequence consists of the first symbol only. Subsequence X_2 _begins at the second symbol. Symbols are added to this subsequence until X_2 _is no longer an element of the vocabulary v{(X_1_X_2_)_π_}. When this occurs, X_2 _is complete, and the construction of X_3 _begins. Consecutive symbols are added to X_3 _until X_3 _∉ v{(X_1_X_2_X_3_)_π_}. The construction of X_4 _then begins. This procedure continues until the entire message is expressed as a concatenation of N subsequences, M = X_1_X_2 _......X_N_. The Lempel-Ziv complexity is the integer N, C_LZ _= N.

This process can be illustrated by a specific example. Suppose M = 000110100.

M = X_1_X_2_X_3_X_4 _and C_LZ _= 4. On reflection it can be seen that this decomposition will provide a mechanism for compressing messages. For any J, by construction subsequence (X_J_)_π _appears somewhere earlier in the message. X_J _can therefore be completely specified by three quantities:

1. the index of the position earlier in the message where (X_J_)_π _begins,

2. the length of X_J_, and

3. the identity of the last symbol of X_J_.

When very large messages are analyzed, X_J _can be very long, perhaps thousands of symbols. This very long substring can now be replaced by these three quantities. Additional examples and pseudo-code for calculating the Lempel-Ziv complexity are given in Appendix A of Watanabe, et al. [163].

**Figure 12:**
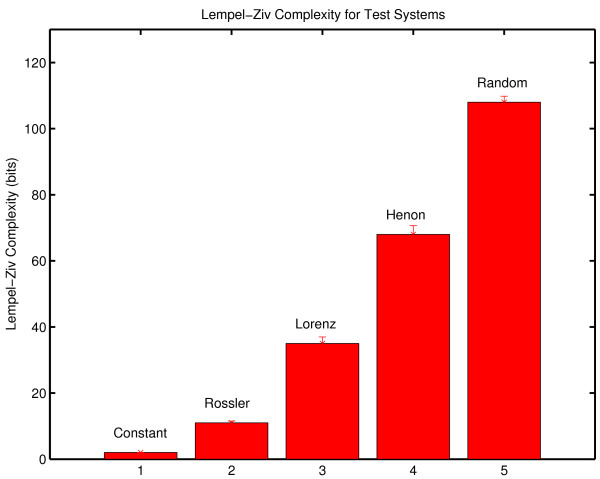
**Lempel-Ziv complexity for one thousand symbol sequences generated by model systems**. Symbol sequences were generated by a binary partition about the median.

The grammar complexity calculations with data generated by model systems reported in Appendix One were repeated with Lempel-Ziv complexity. The same relative ordering was observed random > Hénon > Lorenz > Rössler > constant (Figure 12).

**Figure 13:**
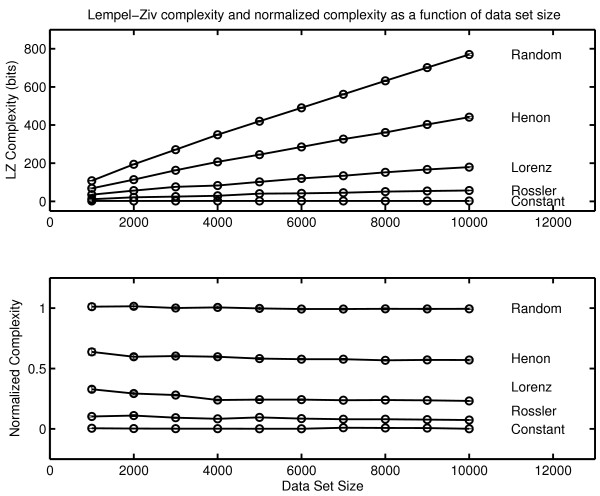
**Lempel-Ziv complexity and normalized complexity as a function of data set size**. Symbol sequences were generated from the previously defined model systems using a binary partition about the median. The upper panel shows Lempel-Ziv complexity. The lower panel shows the corresponding normalized complexity where values of Lempel-Ziv complexity are normalized against random, equi-probable surrogates. In these calculations 499 surrogates were used to compute C_N _(modified from Rapp, 2007).

As shown in Figure 13, Lempel-Ziv complexity shows the same dependence on message length that was observed with grammar complexity. The normalization with equi-probable random surrogates was also implemented with Lempel-Ziv complexity. As before, the normalized complexity is independent of L_M_. In these calculations, 499 surrogates were computed and the null hypothesis was rejected with probability P_NIULL _= .002 in all cases with the exception of random data where the average value of P_NIULL _was .450.

As previously reported [169] grammar complexity and Lempel-Ziv complexity are highly correlated. The complexity values computed with Rössler, Lorenz, Hénon and random data for L_M _= 1000, 2000, .... 10,000 element data sets were compared. The Pearson linear correlation coefficient was found to be r = .998 (the same value that was obtained with different data in [169]). The probability of the null hypothesis of no correlation was less than 10^-8^.

## Appendix Three: Specification of Model System

Five model systems are investigated in the calculations presented in Appendices One and Two. The constant symbol sequence is constructed by repeating one symbol for the entire length of the data set. The Rössler system [173] is a three dimensional system of autonomous ordinary differential equations.

The differential equations were integrated with a step length of h = .1 using a sixth order Runge-Kutta-Hutta algorithm [174]. The Lorenz system [175,176] is specified by

As in the case of the Rössler equations, a Runge-Kutta-Hutta calculation was performed with h = .1. The Hénon system [177,178] is a two dimensional difference equation.

The random number generator [179] produced uniformly distributed random numbers on [0,1]. It is based on L'Ecuyer's two-sequence generator [180] and incorporates a Bays-Durham shuffle [181].

## Pre-publication history

The pre-publication history for this paper can be accessed here:

http://www.biomedcentral.com/1471-244X/11/119/prepub
